# Reconstitution and Structural Analysis of a HECT Ligase-Ubiquitin Complex via an Activity-Based Probe

**DOI:** 10.1021/acschembio.1c00433

**Published:** 2021-08-17

**Authors:** Rahul M. Nair, Ayshwarya Seenivasan, Bing Liu, Dan Chen, Edward D. Lowe, Sonja Lorenz

**Affiliations:** † Rudolf Virchow Center for Integrative and Translational Bioimaging, University of Würzburg, 97080 Würzburg, Germany; ‡ Max Planck Institute for Biophysical Chemistry, 37077 Göttingen, Germany; § Department of Biochemistry, University of Oxford, Oxford, OX13QU, United Kingdom

## Abstract

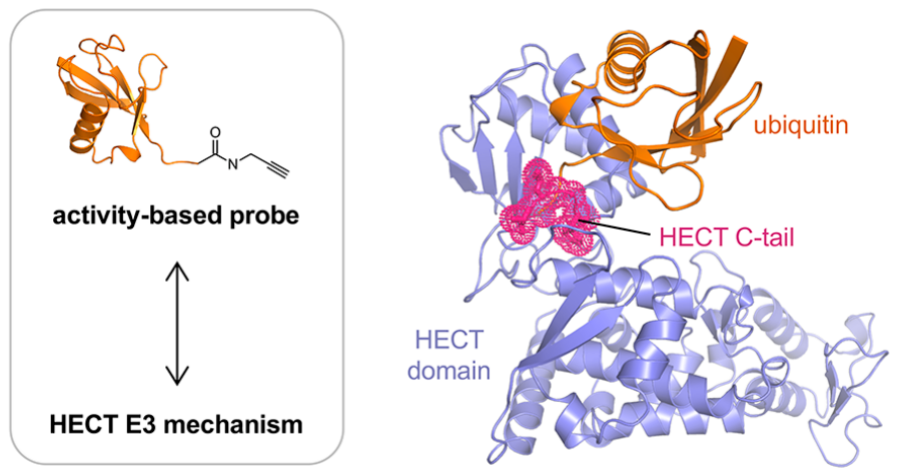

Ubiquitin activity-based probes have proven invaluable in elucidating structural mechanisms in the ubiquitin system by stabilizing transient macromolecular complexes of deubiquitinases, ubiquitin-activating enzymes, and the assemblies of ubiquitin-conjugating enzymes with ubiquitin ligases of the RING-Between-RING and RING-Cysteine-Relay families. Here, we demonstrate that an activity-based probe, ubiquitin-propargylamine, allows for the preparative reconstitution and structural analysis of the interactions between ubiquitin and certain HECT ligases. We present a crystal structure of the ubiquitin-linked HECT domain of HUWE1 that defines a catalytically critical conformation of the C-terminal tail of the ligase for the transfer of ubiquitin to an acceptor protein. Moreover, we observe that ubiquitin-propargylamine displays selectivity among HECT domains, thus corroborating the notion that activity-based probes may provide entry points for the development of specific, active site-directed inhibitors and reporters of HECT ligase activities.

## Introduction

Post-translational modifications of proteins with ubiquitin regulate an astounding range of cellular pathways. This versatility originates largely from the modifier, ubiquitin, being a protein itself, thus holding exquisite regulatory potential through protein–protein interactions and post-translational modifications, including ubiquitin chain formation. Ubiquitination reactions are driven by the sequential formation and reorganization of protein complexes:^1^ A ubiquitin-activating enzyme (E1) generates a thioester linkage between an internal catalytic cysteine and the C-terminal carboxyl group of ubiquitin. A ubiquitin-conjugating enzyme (E2) subsequently takes over ubiquitin by trans-thioesterification and cooperates with a ubiquitin ligase (E3) to link ubiquitin to a primary amino or hydroxyl group of a substrate. E3s in the HECT (Homologous to E6AP C-Terminus), RBR (RING-Between-RING), and RCR (RING-Cysteine-Relay)^2^ families do so via catalytic cysteines that mediate additional trans-thioesterification steps. In contrast, RING (Really Interesting New Gene) E3s facilitate direct ubiquitin transfer from an E2 to a substrate. The actions of ubiquitin ligases are counteracted by deubiquitinases (DUBs) that remove or edit ubiquitin modifications.

To understand the mechanistic underpinnings of this dynamic system requires reconstitution and structure determination of the underlying protein complexes. This has historically been challenging, because of the weak nature of many functionally critical, noncovalent interactions driving ubiquitination and the hydrolytic susceptibility of the thioester linkage between ubiquitin and the E1, E2, and E3, respectively. However, protein engineering combined with enzymatic, semisynthetic, and chemical cross-linking proved successful in stabilizing key intermediates, allowing for their structural visualization.^3^ In particular, ubiquitin activity-based probes (ABPs),^4,5^ which carry an electrophilic warhead to stably link to an enzyme’s active site rather than being processed by it, provided insight into the structural mechanisms of DUBs, E1s, E2-RBR, and E2-RCR ligase complexes.^3^ Surprisingly, while ABPs react with some HECT E3s,^4,6,7^ they have not been applied to structural analyses of this disease-associated, yet therapeutically underexplored E3 class.

Here, we employ ubiquitin-propargylamine (Ub-Prg)^7,8^ to reconstitute and structurally characterize the interaction between the human HECT ligase HUWE1 and active site-linked “donor” ubiquitin. HUWE1 regulates diverse cellular processes, including protein quality control, DNA repair, and transcription; its deregulation is linked to tumorigenesis and neurodevelopmental disorders, with disease-associated mutations accumulating in the C-terminal catalytic HECT domain.^9^ This ∼45-kDa domain consists of an N-terminal lobe (N-lobe), which recruits the ubiquitin-loaded E2, and a C-terminal lobe (C-lobe), which harbors a catalytic cysteine, interacts with the donor ubiquitin, and can encode specificity in isopeptide linkage formation between the donor and an acceptor ubiquitin.^10^ A flexible interlobe linker enables rearrangements of the HECT domain during catalysis: for ubiquitin transfer from the E2 to the E3, the lobes adopt a particular “inverted T” conformation,^11^ whereas the transfer of ubiquitin from the E3 to a substrate requires an “L”-conformation with specific interlobe contacts.^12^ Enabled by the preparative labeling of the HUWE1 HECT domain (HUWE1^HECT^) with Ub-Prg, we define key structural determinants of this L-conformation. Moreover, our findings support the intriguing notion that the selectivity of ABPs for particular HECT domains may be exploited for the development of mechanism-based tools to interrogate or manipulate HECT ligase activities with specificity.

## Results and Discussion

### Ub-Prg Displays Selectivity among Purified HECT Domains

**Figure 1:**
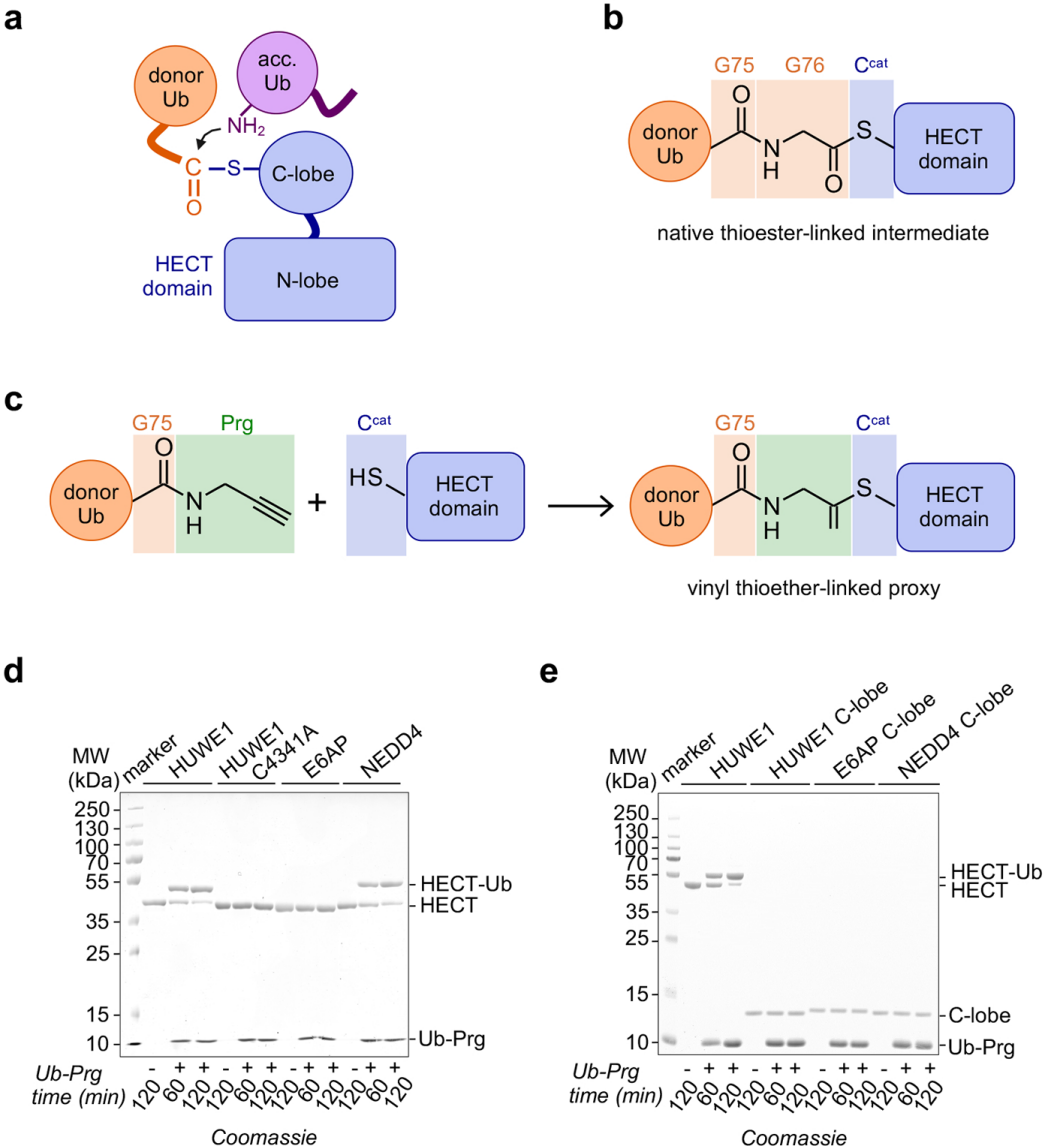
Reactivity of Ub-Prg toward HECT domains: (a) Cartoon of HECT domain-catalyzed ubiquitin linkage formation, where a primary amino group of an acceptor (acc.) ubiquitin (Ub) nucleophilically attacks the C-terminal carbonyl group of a donor ubiquitin, which is thioester-linked to the catalytic cysteine (C^cat^) of the HECT domain; (b) native complex between the donor Ub and a HECT domain; (c) reaction of Ub-Prg with a HECT domain (the chemical formulas in panels (b) and (c) were drawn with ACD/Chemsketch^14^; note that only the carbonyl group of Gly75 is shown). (d, e) Reactivity of Ub-Prg toward different HECT domains (panel (d)) and C-lobes (panel (e)).

A way of stabilizing a donor ubiquitin-HECT E3 complex for structural studies is to replace the native thioester with an engineered disulfide bond.^13^ This strategy typically requires the removal of other reactive cysteines of the E3 in order to achieve specificity for the active site. In the case of HUWE1^HECT^, however, the substitution of surface-exposed, noncatalytic cysteines impairs ligase activity (see Supplementary Figure 1 in the Supporting Information).^4^ We thus interrogated the reactivity of wild-type (WT) HUWE1^HECT^ toward Ub-Prg, which carries an alkyne functionality at the C-terminus of Gly75 and was expected to preferentially react with the active site due to ubiquitin-mediated interactions.^7,8^ The resulting vinyl thioether linkage closely mimics the native thioester, except for one oxygen atom, and is resistant to hydrolysis (Figures 1a–c). Comparative analyses showed that the phylogenetically relatively closely related HECT domains of HUWE1 and NEDD4 readily react with Ub-Prg, while the HECT domain of E6AP does not (Figure 1d). Although both reactive HECT domains have several surface-exposed cysteines, a single product containing one ubiquitin moiety was formed. Substitution of the catalytic Cys4341 of HUWE1 by alanine resulted in a loss of Ub-Prg labeling, confirming that the modification is specific to the active site and recapitulates the donor ubiquitin.

The C-terminal region (“C-tail”) of the HECT domain determines the activity, specificity, and donor ubiquitin binding capacity of various HECT ligases.^12,13,15−19^ Interestingly, the C-tail also influences HECT domain reactivity toward Ub-Prg: a chimeric E6AP HECT domain containing six C-terminal residues from HUWE1 displayed weak Ub-Prg labeling, in contrast to the unreactive WT (see Supplementary Figure 2 in the Supporting Information). This indicates that the C-tail contributes to the reactivity of the active site or ubiquitin binding, while not being the sole determinant. Consistently, HECT domain reactivity toward Ub-Prg requires the N-lobe, as the isolated C-lobes of HUWE1 and NEDD4 were not labeled (Figure 1e). The C-tail and the N-lobe thus cooperate in shaping the active-site environment or Ub-Prg recruitment.

### Crystal Structure of a Vinyl Thioether-Linked Ubiquitin-HECT Domain Complex

**Figure 2:**
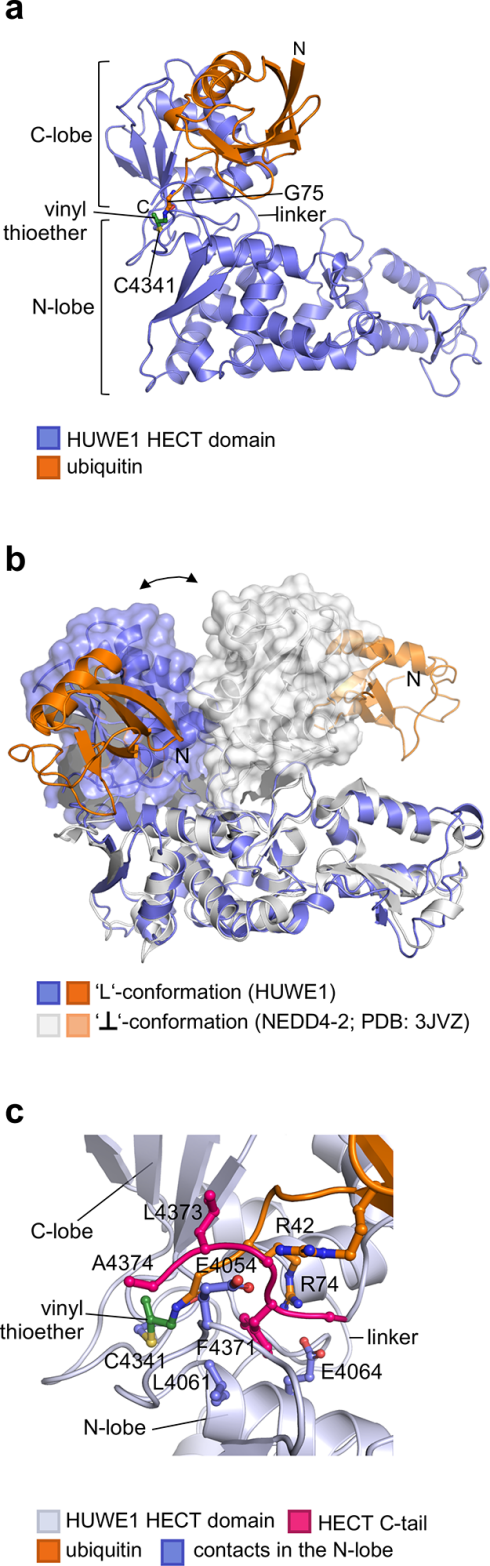
Crystal structure of a Prg-mediated donor ubiquitin-HUWE1^HECT^ complex: (a) crystal structure of the ubiquitin–HUWE1^HECT^ complex in cartoon representation. The vinyl thioether linkage and Gly75 are shown as balls and sticks. C4341 = C^cat^; (b) crystal structures of two donor ubiquitin–HECT domain complexes (this study and PDB: 3JVZ(11)), superposed on the N-lobe (the arrow indicates flexibility of the C-lobe, shown in surface representation); (c) details of the active-site region from panel (a), featuring the side chains of C-tail residues and key contacting residues of the N-lobe and ubiquitin.

To illuminate how the HECT domain of HUWE1 interacts with the donor ubiquitin and understand the significance of the C-tail, we determined a crystal structure of the reconstituted ubiquitin-HUWE1^HECT^ complex (see Table 1). Consistent with our mutational analyses, this structure shows the ubiquitin C-terminus linked to the catalytic cysteine of HUWE1 via a vinyl thioether (see Figure 2a, as well as Supplementary Figure 3(a) in the Supporting Information). The globular portion of ubiquitin contacts the C-lobe through a conserved, catalytically important interface.^11,13,16,17^ Besides this in*-cis* interaction, the crystal lattice does not contain any hydrophobic protein interfaces suspected to be functionally relevant; and, although Cys4184 mediates an intermolecular disulfide bond between adjacent HECT domains in the crystal, the ubiquitin-HUWE1^HECT^ complex is monomeric in solution (see Supplementary Figure 3(b) in the Supporting Information).

**Table 1:** X-ray Crystallographic Data Collection and Refinement Statistics^a^

parameter	value
**Data Collection**	
wavelength	0.9763 Å
resolution range	79.75–2.30 (2.38–2.30)
space group	*C*121
unit cell parameters	
*a*	140.55 Å
*b*	142.17 Å
*c*	103.51 Å
α	90°
β	129.61°
γ	90°
total reflections	368159 (36205)
unique reflections	69327 (6927)
multiplicity	5.3 (5.2)
completeness	99.7% (99.7%)
mean *I*/σ(*I*)	7.1 (0.8)
Wilson B-factor	51.6 Å^2^
R-pim	0.081 (0.906)
CC1/2	0.995 (0.482)
**Refinement**	
reflections used in refinement	69214 (6917)
reflections used for R-free	1766 (177)
R-work	0.23
R-free	0.26
number of non-hydrogen atoms (total)	7562
number of non-hydrogen atoms in macromolecules	7367
number of non-hydrogen atoms in ligands	33
number of non-hydrogen atoms in solvent	162
number of protein residues	918
RMSD of bond lengths	0.004 Å
RMSD of bond angles	0.55°
Ramachandran favored	97.36%
Ramachandran allowed	2.64%
Ramachandran outliers	0%
rotamer outliers	0.13%
clash score	2.68
average B-factor	59.3 Å^2^
average B-factor for macromolecules	59.3 Å^2^
average B-factor for ligands	85.0 Å^2^
average B-factor for solvent	56.8 Å^2^

a Values in parentheses: highest-resolution shell; RMSD = root-mean-square deviation.

Crystal structures of *apo* HUWE1^HECT^^19,18^ show the lobes in an inverted-T conformation. In contrast, our structure of the donor ubiquitin-HUWE1^HECT^ complex reveals an L-conformation (Figure 2b), as seen in distinct crystal forms of several NEDD4-type E3s in *apo*, ubiquitin and substrate-bound forms,^12,20−22^ and structures of a truncated E6AP HECT domain.^23^ Note that the terms “inverted-T” and “L”, in principle, describe solely the relative position of the C-lobe along the long axis of the N-lobe, while each subsumes various rotational states of the C-lobe around the interlobe linker. Interestingly, our crystal structure closely recapitulates the specific rotational state of the C-lobe within an L-conformation that is critical for ubiquitin transfer to a substrate (see Supplementary Figure 4(a) in the Supporting Information). The significance of this architecture was derived from a structure of an Rsp5 construct cross-linked to the donor ubiquitin and a substrate peptide.^12^ The reoccurrence of the same architecture in donor ubiquitin-bound HUWE1^HECT^ supports the idea that it is neither enzyme-specific nor induced by crystal packing, but reflects an inherent, low-energy conformation. Moreover, the observation of both an inverted-T and the functionally relevant L-conformation for the HECT domain of HUWE1 corroborates the notion that they are generally accessible states in the conformational cycle of HECT domains, beyond the NEDD4 subfamily.

### First View of the C-tail of a HECT Ligase in an Active Conformation Required for Isopeptide Bond Formation

**Figure 3:**
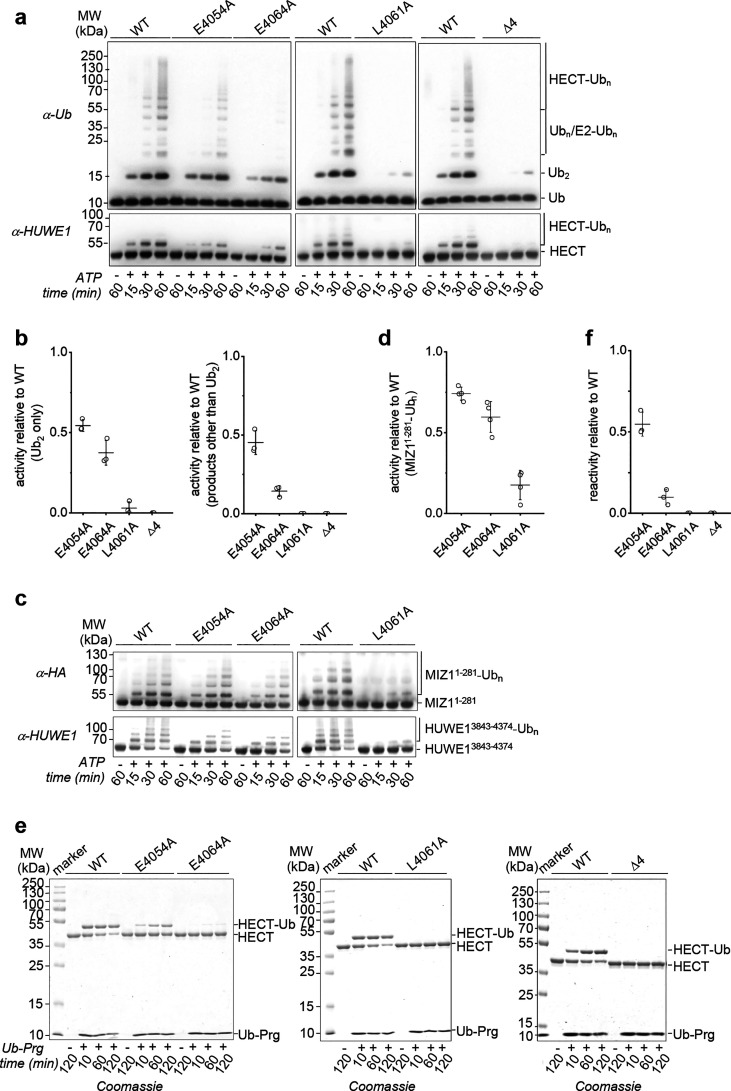
Coordination of the C-tail is required for ligase activity and reactivity toward Ub-Prg. (a) Representative assay analyzing mutational effects at sites coordinating the C-tail in the ubiquitin-HUWE1^HECT^ complex and C-tail truncation (“Δ4”), respectively, on catalytic activity. (b) Quantification of products after 30 min, based on assays as in panel (a); diubiquitin (Ub_2_) and other products (longer chains (Ub_n_; n>2), E2 ubiquitination (E2-Ub_n_; n≥1), and E3 autoubiquitination (HECT-Ub_n_; n≥1)) were quantified separately due to differences in intensity and normalized to the HUWE1 input (bottom blot in panel (a); – ATP); WT activity = 1. (c) Representative assay analyzing mutational effects as in panel (a) on the ubiquitination of HA-tagged MIZ1 (1–281) (MIZ^1-281^-Ub_n_; n≥1) by HUWE1 (3843–4374). (d) Quantification of products after 15 min, based on assays as shown in panel (c); Ubiquitinated MIZ1 was quantified and normalized to the HUWE1 input (bottom blot in panel (C); – ATP); WT activity = 1. (e) Representative assay analyzing mutational effects as in panel (a) on HUWE1^HECT^ reactivity toward Ub-Prg. (f) Quantification of assays as in panel (e). Ubiquitin-labeled HUWE1^HECT^ was quantified and normalized to the HUWE1 input (−Ub-Prg); WT labeling efficiency = 1.

HECT domain-driven ubiquitin transfer to a substrate critically depends on the C-tail.^12,13,15−19^ The structural basis of this requirement, however, has remained elusive, since the C-tail was disordered in previous HECT domain structures. An exception is a structure of an autoinhibited C-terminal construct of HUWE1, in which the C-tail is locked in an inactive state.^18^ Intriguingly, our structure of the ubiquitin-HUWE1^HECT^ complex shows a fully resolved C-tail in a distinct conformation, coordinated by residues of the N-lobe and the C-terminal region of ubiquitin (see Figure 2c, as well as Supplementary Figure 4(b) in the Suppporting Information). Specifically, Phe4371 (−4 position) anchors the C-tail on the N-lobe through hydrophobic contacts with Leu4061. The backbone of the C-tail is embedded by electrostatic interactions at the N-lobe-C-lobe-ubiquitin interface, including Glu4054 and Glu4064 of HUWE1 as well as Arg42 and Arg74 of ubiquitin. To evaluate whether this conformation is catalytically relevant, we replaced the identified contact sites individually with alanine (E4054A, L4061A, E4064A) and monitored HECT domain-mediated isopeptide bond formation. As a negative control, we used a catalytically impaired HECT domain variant lacking four C-terminal residues (“Δ4”).^4,15^ Consistent with our structure-based predictions, the mutated protein variants displayed reduced autoubiquitination and ubiquitin chain formation activities, compared to the WT; alteration of the hydrophobic anchor point, Leu4061, abolished activity almost completely (see Figures 3a and 3b). The same trend was observed for the mutational effects on HUWE1 (residues 3843–4374) activity toward a substrate, the MYC-interacting zinc-finger protein 1 (MIZ1; residues 1–281)) (see Figures 3c and 3d). None of the mutations compromised the structural integrity of the HECT domain, as demonstrated by circular dichroism (CD) (see Supplementary Figure 5 in the Supporting Information). These analyses confirm that the anchoring of the C-tail within the L-conformation is required for ligase activity. Notably, this conformation of the C-tail is compatible with the architecture of the cross-linked Rsp5-donor ubiquitin-substrate peptide complex, in which the C-tail could not be modeled.^12^ Leu4061 and Glu4064 of HUWE1, that are located in a conserved α-helix, are structurally equivalent to Val499 and Glu502 of Rsp5, respectively (see Supplementary Figure 4(a)). Glu4054 is also conserved in Rsp5 (Glu492), but resides in a flexible loop that was not modeled in the crystal structure of the ternary complex. That this loop indeed engages in catalysis, however, is supported by the deleterious effect of a mutation, D495A, in *Rsp5* on substrate ubiquitination.^12^

Consistent with the identified conformation of the C-tail being broadly relevant for the activity of HECT ligases, its contact sites on the N-lobe are rather highly conserved in the HECT E3 family (particularly a hydrophobic residue at the homologous position of Leu4061 and a negatively charged residue at the homologous position of Glu4064 in HUWE1; see Supplementary Figure 6 in the Supporting Information). How the C-tail precisely contributes to ubiquitin ligation is a key open question. It is conceivable that its coordination at the ternary interface of the C-lobe, N-lobe, and ubiquitin tail stabilizes the critical L-state within the conformational equilibrium of the HECT domain. Moreover, intriguingly, this catalytic architecture positions the C-terminus of HUWE1 in close proximity of the catalytic center, which may confer a direct role in catalysis, as previously speculated.^12,13^ In analogy to E2/RING E3-mediated isopeptide bond formation, which is stimulated by an acidic group at the catalytic center of the E2,^24,25^ the C-terminal carboxylate of HECT E3s may contribute to the activation of a lysine nucleophile on an acceptor protein.

### Ub-Prg Reactivity Requires Coordination of the C-Tail in the L-Conformation

Unexpectedly, the C-tail-coordinating residues in the N-lobe do not only determine ubiquitin ligation, but also contribute to the reactivity of HUWE1^HECT^ toward Ub-Prg: reduced labeling was detected for the E4054A and E4064A variants, compared to the WT, and no labeling for L4061A and Δ4 (see Figures 3e and 3f). Consistent with the above analyses, this indicates that the active-site reactivity and/or recruitment of Ub-Prg critically depends on the conformation of the C-tail. The data also suggest that the ABP may preferentially react with the L-conformation, thus exploiting interactions that normally occur during ubiquitin transfer to a substrate. If so, the reaction of Ub-Prg with HUWE1^HECT^ would be mechanistically distinct from E2-ubiquitin conjugate-based probes, which recapitulate the preceding thioesterification step.^6,26^

This study demonstrates the applicability of ubiquitin ABPs for the reconstitution and structural analysis of HECT domain complexes. Although members of this ligase family are tightly linked to human disease, efforts to therapeutically target their catalytic activity have been stalling.^27^ This is largely due to our insufficient understanding of how the intermolecular and intramolecular interactions of the HECT domain influence its conformational dynamics and functions. ABPs may fill this gap by stabilizing critical protein assemblies and guiding the design of specific reporters or inhibitors of HECT ligase activities.

## Methods

### DNA Constructs

The plasmids encoding the HECT domain of HUWE1 (residues 3993–4374)^18^ and E6AP (495–852);^16^ the extended^19^ (3843–4374; for MIZ1 ubiquitination *in vitro*(18)) version of HUWE1^HECT^; the C-lobe of E6AP (741–852);^29^ C-terminally HA-His_6_-tagged MIZ1 (1–282; containing ubiquitination sites);^28^ UBE2L3, UBA1, and ubiquitin^16,18^ were previously described. The intein-chitin-binding domain (CBD)-tagged ubiquitin plasmid was kindly provided by David Komander. The coding sequences for the HECT domain of NEDD4-1 (514-900), the C-lobe of NEDD4-1 (782-900), and the C-lobe of HUWE1 (4255–4374) were cloned into a pET-28a vector (Merck), modified to encode an N-terminal HRV-3C protease-cleavable His_6_-tag. Ligation-free methods were used for all sub-cloning and mutagenesis.

### Protein Preparation

The HECT domain of HUWE1, NEDD4, and E6AP, respectively, was purified from*E*. *coli* LOBSTR RIL (Kerafast) by nickel-affinity and size-exclusion chromatography (SEC) following published strategies.^16,18^ The respective C-lobes, UBA1, UBE2L3, and ubiquitin were prepared as described,^16,29^ likewise MIZ1 (1–282)^28^. The preparation of the ubiquitin-HUWE1^HECT^ complex was also guided by published protocols.^30^ Specifically, intein-CBD-tagged ubiquitin was expressed in*E*. *coli* BL21(DE3) at 20 °C overnight upon induction with 0.5 mM IPTG; the cleared lysate incubated with chitin resin (NEB) in 50 mM HEPES, 100 mM NaCl, pH 8.0 (buffer 1) at 4 °C for 5 h, the resin washed with buffer 1, protein released with 150 mM sodium 2-mercaptoethanesulfonate (Sigma–Aldrich), dialyzed at 4 °C overnight into buffer 1, incubated with 150 mM Prg (Sigma–Aldrich) at RT for 5 h, dialyzed again at 4 °C overnight into buffer 1, and subjected to SEC (Superdex (SD) 75 16/600 GL column; GE Healthcare) in buffer 1. Ub-Prg was incubated with purified HUWE1^HECT^ at a 5:1 molar ratio and 30 °C overnight, followed by SEC (SD 75 16/600 GL) in 20 mM HEPES, 150 mM NaCl, 1 mM EDTA, 5 mM DTT, pH 8.0.

### ABP Reactions

10 μM E3 and 100 μM Ub-Prg were incubated in 50 mM HEPES, 100 mM NaCl, pH 8.0 at 30 °C, quenched with SDS loading dye at the indicated times, and analyzed by SDS PAGE with Coomassie staining.

### X-ray Crystallography

The ubiquitin-HUWE1^HECT^ complex crystallized at 10 mg mL^–1^ and 20 °C upon streak seeding in sitting drops containing 0.65 M sodium phosphate monobasic, potassium phosphate dibasic, 0.1 M HEPES, pH 7.5. Crystals were cryo-protected in the same solution including 20% (v/v) glycerol; diffraction data collected at beamline P14, PETRA III (DESY) and processed with XDS.^31^ Molecular replacement was performed with Phaser,^32^ using N-lobe (3993–4256) and C-lobe (4257–4366) structures extracted from PDB3H1Das search models. Refinement was performed with Phenix^33^ using individual B-factors; model building with Coot.^34^ Electron densities were rendered with phenix.maps^33^ and structures with PyMOL (open source, V1.7.6; DeLano Scientific LLC).

### Enzymatic Assays

To monitor isopeptide bond formation independent of substrate, 200 nM UBA1, 5 μM UBE2L3, 5 μM HUWE1^HECT^ variants, and 100 μM ubiquitin were incubated with 3 mM ATP and 8 mM MgCl_2_ in 25 mM HEPES, pH 7.4 at 30 °C. For substrate ubiquitination, 200 nM UBA1, 5 μM UBE2L3, 5 μM HUWE1 (3843–4374) variants, 100 μM ubiquitin, and 12 μM MIZ1 (1–282) were incubated under the same conditions. Reactions were quenched with SDS loading dye at the indicated times and analyzed by SDS-PAGE and Western blotting with anti-ubiquitin P4D1 (Santa Cruz Biotechnology), anti-HUWE1 (SAB2900746; Sigma–Aldrich), or anti-HA C29F4 (Cell Signaling Technology) antibodies.

### Quantification

Reaction input and products were quantified with ImageJ;^35^ the mean and SDs from three independent experiments were plotted with OriginPro 2020 (OriginLab).

### CD

Fifteen spectra of 2.5 μM protein in 50 mM potassium phosphate, 50 mM sodium phosphate, pH 7.5 were accumulated at 20 °C with a JASCO J-810 spectropolarimeterin a 0.01 cm quartz cuvette (0.1 nm steps; 190–260 nm; 20 nm min^–1^; 1 nm bandwidth), the buffer spectrum was subtracted, and the molar ellipticity, [Θ], calculated.^36^

### SEC MALS

150 μg of the ubiquitin-HUWE1^HECT^ complex was analyzed at RT with an SD 75 10/300 GL column (GE Healthcare) coupled to Dawn8+ and Optilab T-rEX detectors (Wyatt Technology); the data were processed with ASTRA 6 (Wyatt Technology).

## Supporting Information Available

Supplementary Figures 1–6 and supplementary references (PDF)

## Accession Codes

Atomic coordinates and structure factors were deposited in the Protein Databank (PDB), accession code6XZ1.
