# Supplementary material for: Reconstitution and Structural Analysis of a HECT Ligase-Ubiquitin Complex via an Activity-Based Probe
Source: ACS Chem Biol. 2021 Aug 17;16(9):1615–21. doi: 10.1021/acschembio.1c00433 (PMC8453484; doi:10.1021/acschembio.1c00433)
Supplement: Supplementary file 1 — cb1c00433_si_001.pdf [file cb1c00433_si_001.pdf]

SUPPLEMENTARY INFORMATION

Reconstitution and structural analysis of a HECT ligase-ubiquitin complex via an activity-based probe

Rahul M. Nair[1], Ayshwarya Seenivasan[2], Bing Liu[1], Dan Chen[1], Edward D. Lowe[3], and Sonja Lorenz[2],*

[1]Rudolf Virchow Center for Integrative and Translational Bioimaging, University of Würzburg, 97080 Würzburg, Germany

[2]Max Planck Institute for Biophysical Chemistry, 37077 Göttingen, Germany

[3]Department of Biochemistry, University of Oxford, Oxford, OX13QU, United Kingdom

*correspondence: sonja.lorenz@mpibpc.mpg.de (S.L.)

Supplementary Figure 1

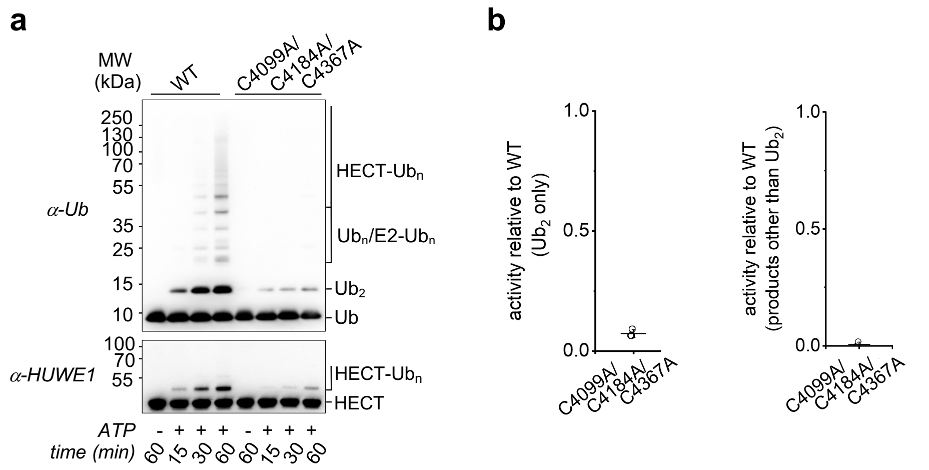

Supplementary Figure 1. Effects of cysteine substitutions on the activity of the HUWE1 HECT domain

a) Representative assay analyzing the effect of replacing three surface-exposed, non-catalytic cysteine residues in HUWE1^{HECT} (‘HECT’) with alanine on ubiquitination activity. Reactions were quenched at the indicated time points and analyzed by SDS PAGE and Western blotting. b) Quantification of reaction products after 30 minutes, based on assays as shown in (a). Di-ubiquitin (Ub_2) and other products (longer chains (Ub_n; n>2), E2 ubiquitination (E2-Ub_n; n ≥1), and E3 auto-ubiquitination (HECT-Ub_n; n≥1)) were quantified separately due differences in intensity and subsequently normalized to the HUWE1 input (bottom blot (a)); WT activity = 1.

Supplementary Figure 2

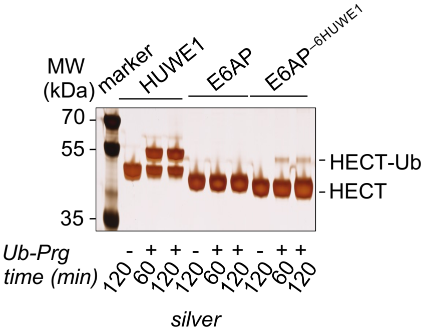

Supplementary Figure 2. Influence of the C-tail on the reactivity of the E6AP HECT domain toward Ub-Prg

Assay monitoring the labeling of the indicated HECT domain variants with Ub-Prg. E6AP$^{–6HUWE1}$ denotes a chimeric version of the E6AP HECT domain containing the six C-terminal residues of HUWE1. Reactions were quenched at the indicated time points and analyzed by SDS PAGE and silver staining in order to detect the weak labeling of the E6AP$^{–6HUWE1}$ variant.

Supplementary Figure 3

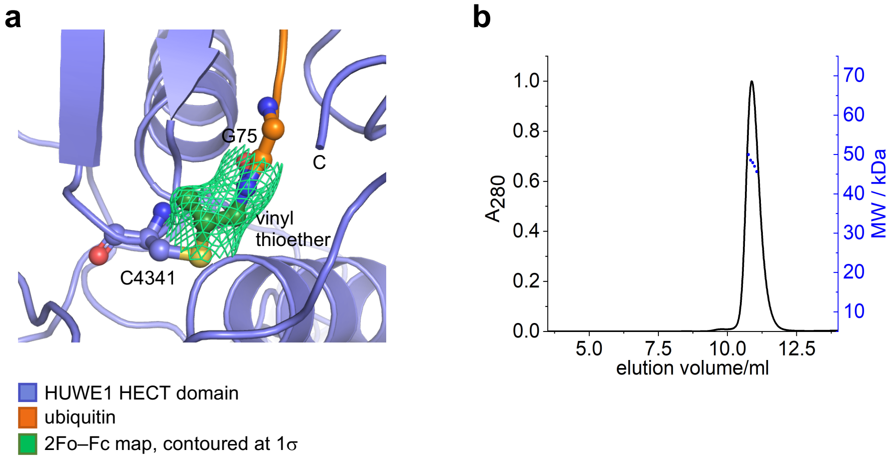

Supplementary Figure 3. X-ray crystallographic features and oligomeric state of the ubiquitin-HUWE1 HECT domain complex

a) Detail of the crystal structure of the ubiquitin-HUWE1^{HECT} complex (this study), focusing on the vinyl thioether linkage between the active-site cysteine of HUWE1 (Cys4341) and the C-terminal residue (Gly75) of the ubiquitin variant, shown as balls-and-sticks. A 2Fo–Fc omit map, contoured at 1σ, is displayed for the vinyl thioether linkage. b) SEC MALS analysis of the purified ubiquitin-HUWE1^{HECT} complex. The UV signal (left y-axis) is shown continuously; the MALS signal (right y-axis; converted to molecular weight (MW)) is displayed across the absorbance peak, as used for MW determination, yielding 56 ± 4 kDa (calculated MW: 53.4 kDa).

Supplementary Figure 4

a

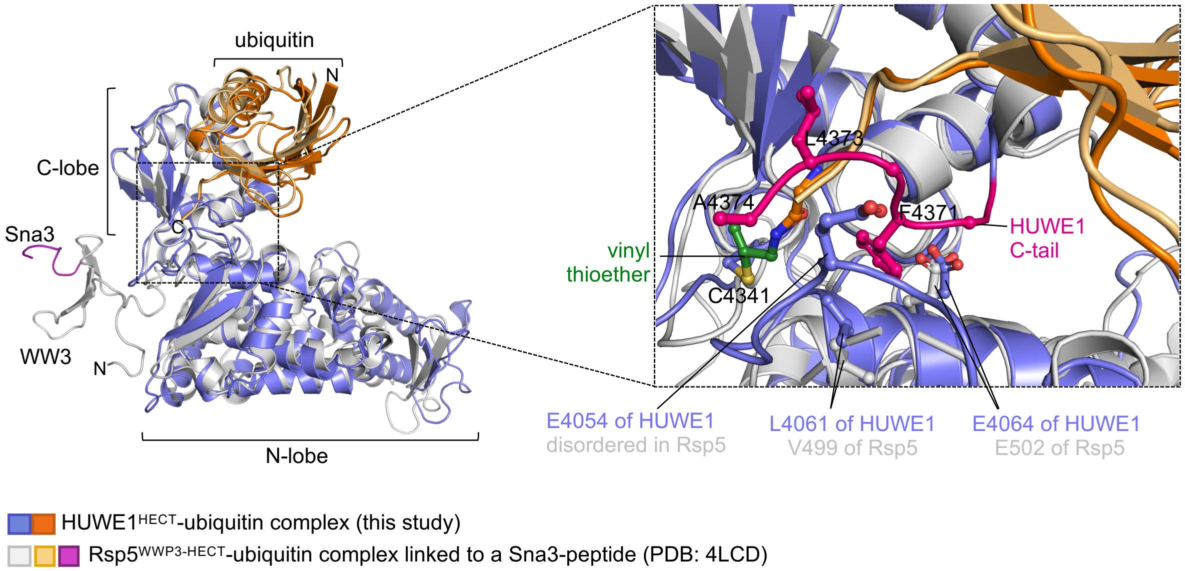

b

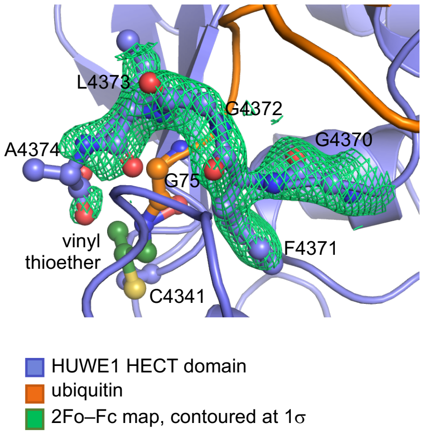

Supplementary Figure 4. Structural features of the ubiquitin-HUWE1 HECT domain complex, focusing on the C-tail

a) Crystal structures of ubiquitin-bound HUWE1^{HECT} (this study) and a crosslinked, ternary complex of a Rsp5 construct containing the WW3 and HECT domain, the donor ubiquitin, and a Sna3-derived substrate peptide (PDB: 4LCD[1]), superposed on the C-lobe (left). Detailed view of the boxed region showing the side chains of the HUWE1 C-tail, key contacting side chains on the N-lobe, homologous side chains thereof in Rsp5, and the vinyl thioether linkage between the donor ubiquitin and C4341 of HUWE1^{HECT}. Note that the ternary crosslinker and surrounding regions in Rsp5 were not modelled, including the region homologous to the Glu4054-containing loop of HUWE1 (right). b) Detail of the crystal structure of the ubiquitin-HUWE1^{HECT} complex (this study), focusing on the C-tail, shown as balls-and-sticks, along with a 2Fo–Fc omit map, contoured at 1σ.

Supplementary Figure 5

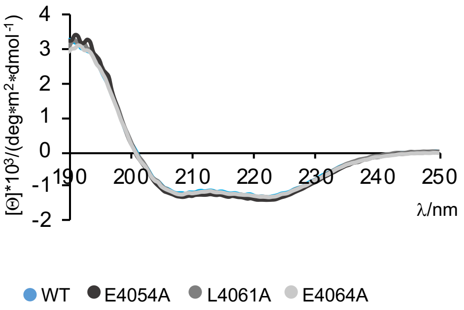

Supplementary Figure 5. Circular dichroism analysis of purified HUWE1 HECT domain variants

Superposition of the CD spectra of WT HUWE1^{HECT} and variants thereof that contain amino acid substitutions at the functionally critical N-lobe-C-tail interface in the crystallized, ubiquitin-bound L-conformation.

Supplementary Figure 6

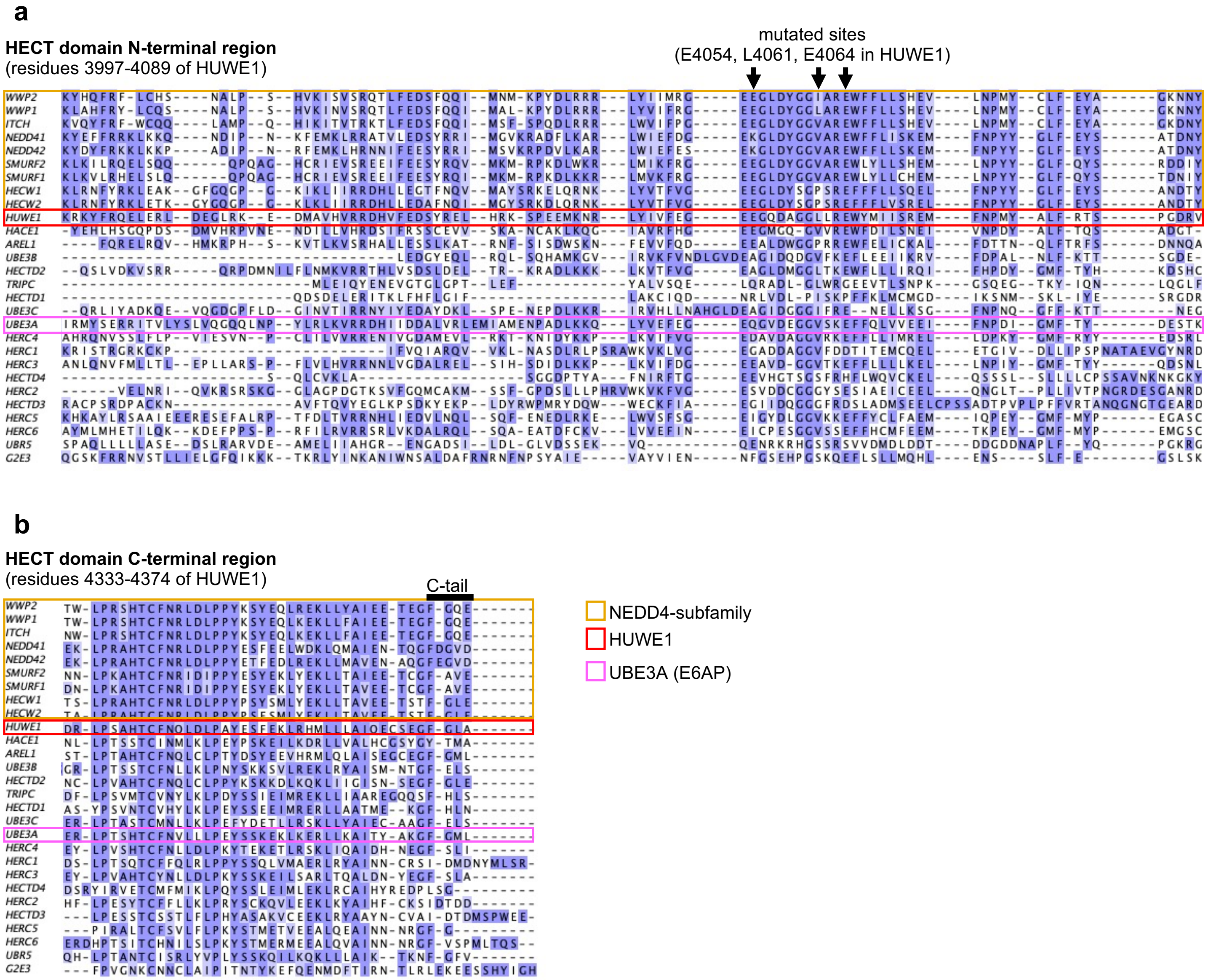

Supplementary Figure 6. Amino acid sequence alignments of selected regions of the human HECT ligases

a, b) The amino acid sequences of the HECT domains of the 28 human HECT ligases were aligned with MUSCLE[2]. Selected regions of this alignment were illustrated with Jalview[3], colored according to the BLOSUM62[4] score. Note that the alignment for the most divergent sequences (e.g., HECTD1 and TRIPC) should be treated with caution.

Supplementary references

(1) Kamadurai, H. B., Qiu, Y., Deng, A., Harrison, J. S., MacDonald, C., Actis, M., Rodrigues, P., Miller, D. J., Souphron, J., Lewis, S. M., Kurinov, I., Fujii, N., Hammel, M., Piper, R., Kuhlman, B., and Schulman, B. A. (2013) Mechanism of ubiquitin ligation and lysine prioritization by a HECT E3. *eLife 2*, e00828–e00828.

(2) Edgar, R. C. (2004) MUSCLE: multiple sequence alignment with high accuracy and high throughput. *Nucleic Acids Res 32*, 1792–1797.

(3) Waterhouse, A. M., Procter, J. B., Martin, D. M. A., Clamp, M., and Barton, G. J. (2009) Jalview Version 2-a multiple sequence alignment editor and analysis workbench. *Bioinformatics 25*, 1189–1191.

(4) Henikoff, S., and Henikoff, J. G. (1992) Amino-acid substitution matrices from protein blocks. *Proceedings of the National Academy of Sciences of the United States of America 89*, 10915–10919.
